# Links Between Testosterone, Oestrogen, and the Growth Hormone/Insulin-Like Growth Factor Axis and Resistance Exercise Muscle Adaptations

**DOI:** 10.3389/fphys.2020.621226

**Published:** 2021-01-15

**Authors:** Nima Gharahdaghi, Bethan E. Phillips, Nathaniel J. Szewczyk, Ken Smith, Daniel J. Wilkinson, Philip J. Atherton

**Affiliations:** Medical Research Council-Versus Arthritis Centre for Musculoskeletal Ageing Research and Nottingham National Institute for Health Research Nottingham Biomedical Research Centre, School of Medicine, University of Nottingham, Derby, United Kingdom

**Keywords:** hormone, resistance exercise, muscle growth, protein synthesis, hypertrophy

## Abstract

Maintenance of skeletal muscle mass throughout the life course is key for the regulation of health, with physical activity a critical component of this, in part, due to its influence upon key hormones such as testosterone, estrogen, growth hormone (GH), and insulin-like growth factor (IGF). Despite the importance of these hormones for the regulation of skeletal muscle mass in response to different types of exercise, their interaction with the processes controlling muscle mass remain unclear. This review presents evidence on the importance of these hormones in the regulation of skeletal muscle mass and their responses, and involvement in muscle adaptation to resistance exercise. Highlighting the key role testosterone plays as a primary anabolic hormone in muscle adaptation following exercise training, through its interaction with anabolic signaling pathways and other hormones via the androgen receptor (AR), this review also describes the potential importance of fluctuations in other hormones such as GH and IGF-1 in concert with dietary amino acid availability; and the role of estrogen, under the influence of the menstrual cycle and menopause, being especially important in adaptive exercise responses in women. Finally, the downstream mechanisms by which these hormones impact regulation of muscle protein turnover (synthesis and breakdown), and thus muscle mass are discussed. Advances in our understanding of hormones that impact protein turnover throughout life offers great relevance, not just for athletes, but also for the general and clinical populations alike.

## Introduction

Skeletal muscle accounts for ~40–45% of total body mass (Romagnoli et al., 2020). Following a rapid post-natal growth phase, skeletal muscle mass is typically maintained at a steady state in adulthood through a controlled balance between muscle protein synthesis (MPS) and breakdown (MPB)—unless in the presence of physiological (exercise) or pathological (age or disease) stimuli. The mitigation of age and disease-related muscle wasting and dysfunction remains a major research effort. Even today after significant efforts to develop pharmaceutical strategies to mitigate muscle wasting (Sepulveda et al., 2015), contractile activity in the form of resistance exercise (RE) remains the most efficacious intervention. RE training (RET) leads to muscle hypertrophy through a sustained elevation of MPS (Hooper et al., 2017)—responses which are driven by a combination of mechanical overload, and an associated release of hormones; e.g., androgens and insulin-like growth factor-1 (IGF-1) (Romagnoli et al., 2020) and by muscle mechano-sensitive signals.

While the fundamental roles of hormones in muscle development and their decline in aging are well-established, the impact of physiological fluctuations (e.g., due to circadian rhythms or transient increases following bouts of RE) in hormones remains unclear (Schroeder et al., 2013). RE induces marked anabolic hormone responses, in particular those involving testosterone, growth hormone (GH) and IGF-1 (Spiering et al., 2008). These hormonal elevations in response to RE take place in a unique physiological environment, whereby acute elevations in circulating blood hormone concentrations, resulting from a combination of either e.g., increased secretion, reduced hepatic clearance, reduced plasma volume or reduced degradation rates—interact with receptors on the target tissue cell membranes or with nuclear/cytoplasmic receptors located within the target tissue (e.g., steroid receptors); which, alongside mechano-signaling, initiates a sequence of molecular events, leading to muscle adaptive responses such as an increase in MPS and/or a decrease in MPB (Kraemer and Ratamess, 2005). Given the apparent complexity of RE-induced hormonal responses and their impact on muscle adaptation, we aim to provide an update on advances in this area.

## Testosterone

**Figure 1:**
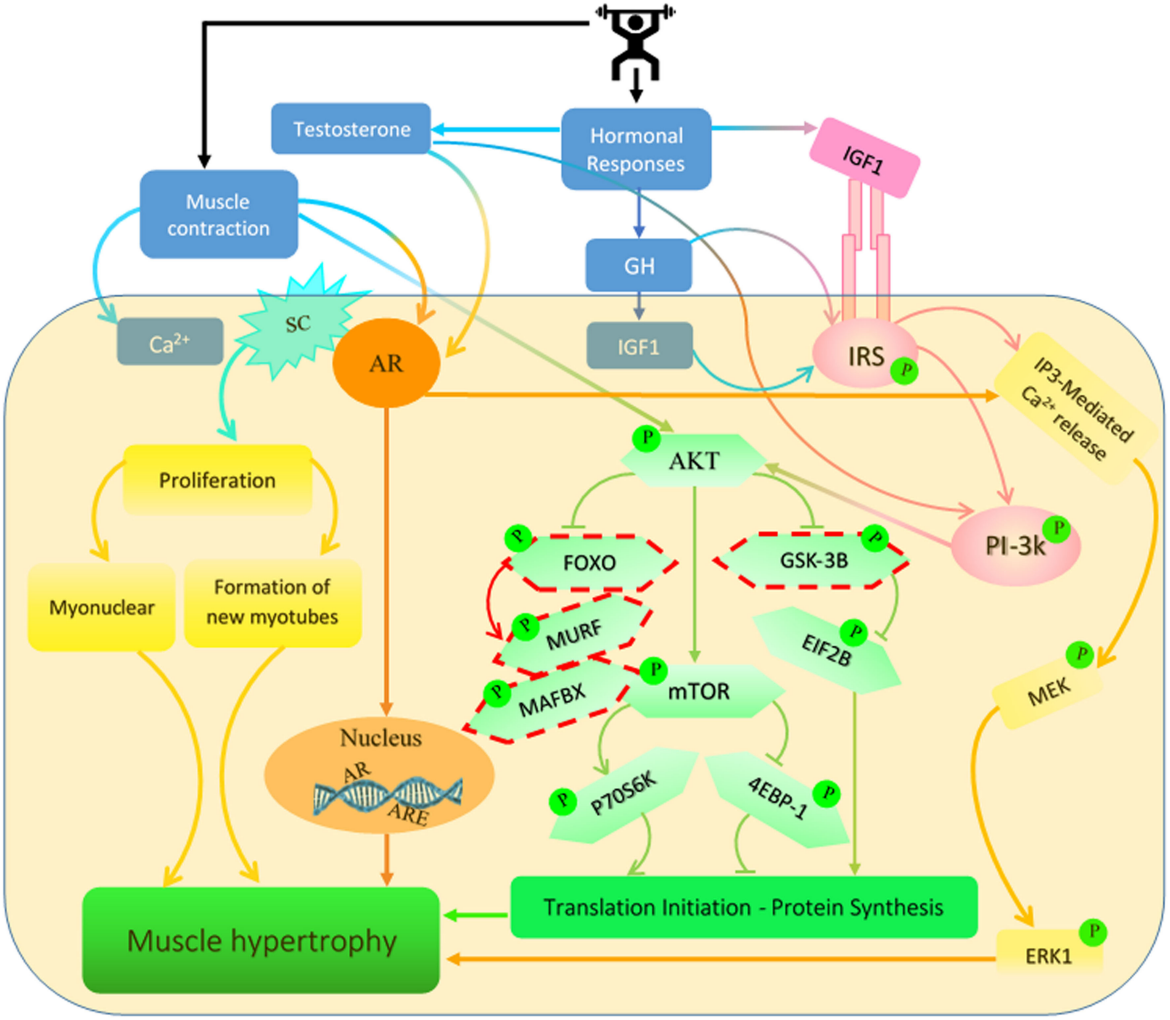
Signaling pathways regulated by testosterone, growth hormone (GH) and insulin-like growth factor 1 (IGF-1) are induced by resistance exercise (RE). RE has been shown to increase the concentration of these hormones which activate several different signaling pathways in the muscle. These pathways lead to increases in muscle protein synthesis (MPS) and net protein accretion which result in an increase in muscle mass. SC, satellite cell; AR, androgen receptor; IRS, insulin receptor substrate; ARE, androgen response element. *Dashed outline represents inhibitory protein cascades.

The principal androgen, testosterone, is an anabolic-androgenic steroid hormone which is synthesized from cholesterol—produced mainly in Leydig cells in men, and the ovary (25%) and adrenalzona fasciculata (25%) in women, via conversion from progesterone, with the remaining ~50% being produced from circulating androstenedione (Burger, 2002). Homeostatic processes maintain systemic testosterone levels within the range of 7.7–29.4 nmol.L^−1^ in healthy young men and 0.1–1.7 nmol.L^−1^ in healthy menstruating women under 40 y (Handelsman et al., 2018). In contrast, there are no differences observed between men and women in relation to intramuscular testosterone concentrations and steroidogenic enzymes (Vingren et al., 2008). Systemic testosterone is taken up by the muscle through its binding to membrane-bound or cytoplasmic androgen receptors (AR), in turn stimulating subsequent myocellular signaling (Vingren et al., 2010; Kraemer et al., 2020) and altering the expression of thousands of genes, many of which are involved in the regulation of skeletal muscle structure, fiber type (Dubois et al., 2014), intramyocellular metabolism (White et al., 2013), and mRNA transcription (MacLean et al., 2008) (Figure 1). Once bound to the AR, testosterone is irreversibly converted to dihydrotestosterone (DHT) through the enzymatic action of 5-α reductase (Wilborn et al., 2010). It is generally accepted that DHT is the more potent hormone due to its receptor binding kinetics (Ly et al., 2001); however, testosterone has also been shown to regulate a multitude of ergogenic, anabolic, and anti-catabolic functions in skeletal muscle, without prior conversion to DHT (Bhasin et al., 2003). Testosterone also effects the development of bone, connective and neural tissues (Hoffman et al., 2009), leading to increased muscle strength, power, endurance, and hypertrophy in a dose-dependent manner (Sinha-Hikim et al., 2006; Kraemer et al., 2017). The importance of androgens for mediating muscle growth are substantiated through numerous lines of evidence, including: (1) that exogenous administration, potentiates gains in muscle strength and muscle mass (Gharahdaghi et al., 2019); (2) that gonadotropin-releasing hormone analogs, which inhibit endogenous testosterone release, prevent gains in muscle strength and attenuate gains in muscle mass (Kvorning et al., 2007); and (3) that AR antagonists, which inhibit endogenous testosterone from binding to the AR, impair radical muscle growth during synergist overload (Inoue et al., 1994). Together, these findings suggest a significant role for testosterone in regulating adult muscle growth in response to mechanical loading (i.e., RE).

### Exercise-Induced Testosterone Release, and Links With Muscle Adaptation by Sex and Age

The relationship between RE and testosterone responses have been extensively reviewed in young men (Ahtiainen et al., 2003, 2005; West et al., 2009; West and Phillips, 2012), with the majority of studies suggesting that it is the *acute* transient elevations in testosterone that likely drive the proposed hormonal adaptations associated with muscle growth. For example, immediately following RE, serum testosterone levels peak [~from 13 (resting levels) to 38 (at ~30 mins) nmol.L^−1^] with a concomitant upregulation of AR mRNA and protein content within the muscle (Willoughby and Taylor, 2004; Hooper et al., 2017). It seems high intensity RE stimulates basophilic cells of the anterior pituitary to release luteinizing hormone (LH) from gonadotrophs in the anterior pituitary which then acts as the primary regulator of testosterone secretion from the Leydig cells of the testes (Fry and Kraemer, 1997). High affinity binding of LH at the Leydig cells of the testes activates a cyclic adenosine mono phosphate (cAMP) mechanism, resulting in increased testosterone synthesis (Dufau and Catt, 1979; Fry and Kraemer, 1997). This activation is dependent on the frequency and amplitude of LH secretion, with pulses occurring in the human at the rate of ~8–14 pulses.day^−1^ in men. Secretion of LH is principally regulated by LH releasing hormone (LHRH) via portal circulation from the hypothalamus. High affinity binding of LHRH in the anterior pituitary activates LH secretion by a calcium-dependent mechanism, resulting in LH secretion (Dufau and Catt, 1979; Fry and Kraemer, 1997). Further, early increased circulating testosterone levels during RE are also LH-independent and it seems they may be directly stimulated via increases in lactate levels induced by an increase in the production of cAMP in testicular tissues (Lin et al., 2001). However, the mechanisms of lactate action on testosterone production by Leydig cells are not clear yet. While the acute response of testosterone returns to baseline rapidly post exercise and has been shown to not be elevated chronically following repeated bouts of RE (Hooper et al., 2017); the acute upregulation of AR mRNA and protein content can last up to 1–2 days post RE (Ratamess et al., 2005), thereby augmenting testosterone uptake into the muscle, and potentiating the anabolic effects of testosterone over longer periods (Murphy and Koehler, 2020; Tinline-Goodfellow et al., 2020). It therefore may be that the combined effects of acute testosterone elevation post exercise and sustained AR upregulation in the muscle may represent an additional mechanism through which RE might regulate muscle growth. Notably, while some studies have indicated correlative relationships between RE-induced elevations in testosterone and muscle strength and hypertrophy (Hansen et al., 2001; Ahtiainen et al., 2003, 2005), this remains equivocal (West et al., 2009; West and Phillips, 2012) perhaps since the magnitude of acute responses in young males can be influenced by many factors e.g., timing of sampling etc. Additionally, there is evidence that RE loads of <70% 1-RM (Tremblay et al., 2004; Yarrow et al., 2007; Fry and Lohnes, 2010; Hough et al., 2011); programs incorporating only upper body exercises, even at a relatively high intensity and volume (Migiano et al., 2010); and those with the long rest periods between repetitions do not stimulate a significant post exercise testosterone response (McCaulley et al., 2009), despite post exercise increases in MPS, anabolic signaling and associated muscle growth adaptive responses being observed (West et al., 2009; West and Phillips, 2012). In an attempt to better understand the discrepancies between testosterone and muscle adaptive responses, Phillips and colleagues devised a unique experimental approach, whereby they compared a “high” vs. “low” hormone environment (induced by working distinct muscle bulk) (West et al., 2010). Despite the contrasting hormonal profiles and significantly different acute testosterone responses in these environments, muscle mass and strength gains were comparable, suggesting that the role of testosterone (and other hormones) in exercise induced muscle adaptation is minimal. Conversely, other studies using similar procedures reported that the “high” hormone environment potentiated muscle strength gains after 9 weeks RET (Hansen et al., 2001) and acute AR content 180 min after RE (Spiering et al., 2009), which was associated with increased MPS, muscle growth and recovery (Sheffield-Moore, 2000). The differences in outcomes between these studies may be driven by the experimental design (different biopsy location; i.e., biceps brachii vs. vastus lateralis and different testosterone inducing exercise regimes, which resulted in different peak testosterone; i.e., 27 (West et al., 2010) vs. 38 nmol.L^−1^ (Spiering et al., 2009) and also the time course of muscle sampling (between 3 and 4 h post-RE). These differences may be important since the duration and magnitude of testosterone and AR elevation and AR exposure to testosterone appear to play a crucial role in skeletal muscle adaptations both *in vitro* (Bloomer et al., 2000) and *in vivo* (Antonio et al., 1999; Ferrando et al., 2002). In turn, as the pituitary-gonadal axis works in a negative feedback loop, increasing AR content will likely result in enhanced tissue uptake of testosterone, thus lowering circulating testosterone. Reductions in circulating testosterone concentrations (due to enhanced cellular uptake) are monitored by the hypothalamus, which releases gonadotropin-releasing hormone (GnRH) to stimulate LH secretion, then testosterone synthesis/secretion (Kraemer et al., 2006). Herein, it is suggested that AR protein content itself may be critical in RE-induced skeletal muscle protein accretion, with AR content, and not circulating testosterone being more closely associated with adaptations in muscle mass (Morton et al., 2018) and fiber CSA (Mitchell et al., 2013) in young men.

Whilst the majority of investigations into the role of testosterone in muscle adaptive response have been performed in males (reflecting male biology), the importance of circulating concentrations of testosterone in adult women should not be underestimated based on its biological role in the conversion of progesterone to the principal oestrogens—oestradiol and oestrone (Cui et al., 2013). That being said, the importance of testosterone in women remains unclear since while there is an indispensable role e.g., on bone health, in older males (Mohamad et al., 2016), a reduction in testosterone generally does not occur independently of other hormones (such as the oestrogens) in females (e.g., following the menopause) (Chakravarti et al., 1976). Moreover, females do not have Leydig cells; the cells which are likely the source of the acute RE-induced increase in testosterone in men (Kvorning et al., 2007). That said, increases in testosterone in females have been reported in response to RE in some (Nindl et al., 2001; Copeland et al., 2002), but not all (Marx et al., 2001; Linnamo et al., 2005) studies, albeit with claims of no, or limited, effects of acute testosterone elevations in relation to muscle growth in women (Kraemer et al., 2017). Therefore, the links between the testosterone response and exercise adaptation in women remain contentious and require further investigation.

Finally, it is important when overviewing the role of testosterone in controlling muscle mass, to consider older adults. With aging, there is a linear decline in bioavailable circulating testosterone in both men and women (Kraemer et al., 1998; Hakkinen et al., 2000), with these reductions leading to osteoporosis in both sexes (Mohamad et al., 2016). Testosterone is ~98% bound to serum proteins (sex hormone-binding globulin (SHBG) and albumin) and only 1–2% of testosterone is unbound or free. Because testosterone is bound to SHBG with high affinity, it is not available to most tissues for action. Given the concentration of SHBG increases across the lifespan in men (Liu et al., 2007) and only increase after ~60 y in women (Maggio et al., 2008), bioavailable testosterone (free plus albumin-bound testosterone) concentrations decline even more markedly than total testosterone levels with aging (Matsumoto, 2002). This reduction can eventually lead to very low resting concentrations of circulating testosterone particularly in men, creating the so-called andropause (Vingren et al., 2010). Intriguingly, this reduction in testosterone tracks with the gradual decline in muscle mass observed with age, i.e., ~1–3% decline in circulating testosterone and 1–2% loss of muscle mass in men (Vingren et al., 2010; Gharahdaghi et al., 2019), perhaps suggesting declines in endogenous testosterone may be linked to loss of muscle mass. Moreover, while RE in older men (> 59 yr old) still elicits an acute elevation in circulating testosterone, the magnitude of elevation is smaller than that of younger men performing the same RE (Kraemer et al., 1998; Hakkinen et al., 2000), i.e., ~1 nmol.L^−1^ in young vs. ~0.1 nmol.L^−1^ in older men (Brook et al., 2016), ostensibly leading to reduced muscle AR content, MPS and ultimately blunted muscle adaptive responses to chronic RET (Brook et al., 2016; Gharahdaghi et al., 2019). With provision of exogenous testosterone helping to restore this blunting somewhat (Gharahdaghi et al., 2019), the influence of testosterone on muscle may be small and permissive in the young, but the need for hormonal input for the control of muscle mass may be more important as we age to overcome age-related deficits in the responsiveness of older muscle to exercise training.

### Metabolic and Molecular Effects of Testosterone in Skeletal Muscle

Anabolic effects of AR and testosterone upregulation after RE occur through a combination of both genomic i.e., transcriptional capacity, and non-genomic i.e., translational efficiency, pathways (Kraemer et al., 2020). RE and androgens up-regulate muscle AR content via distinct mechanisms; RE increases AR mRNA transcription via RhoA (a member of the Rho family of small GTPases which is involved in muscle transcription factor) and serum response factor (MADS-box transcription factor which is essential for muscle-specific gene expression) signaling (Lee et al., 2003); in contrast, testosterone increases muscle AR via enhanced AR mRNA association with polyribosomes, increasing AR mRNA translation (Mora and Mahesh, 1999) and doubling AR half-life from ~3.1 to 6.6 h (Syms et al., 1985). Due to these contrasting mechanisms of action, a combination of RE and RE-induced testosterone secretion will likely potentiate post exercise AR responses for longer, thereby augmenting adaptive muscle growth. Conversely, impaired testosterone responsiveness to RE in older adults, likely attenuates the AR response, due to lack of testosterone mediated AR increases, and subsequently, limits muscle mass gains with RET. When testosterone binds to the AR, the AR transforms, dimerizes and translocates to the nucleus, binding to androgen-response elements (ARE) therein, as a homodimer. Activation of these AREs stimulates the transcription of protein targets and other anabolic systems, such as the local production of IGF-1 which is related to muscle protein accretion through a decrease in IGFBP-4 mRNA concentration coupled with an increase in IGF-1 mRNA (Bamman et al., 2001; West et al., 2010) (see IGF-1 section). In addition to these genomic signaling pathways, testosterone is thought to independently activate the Akt/mTOR/S6K1 pathway, which represents an integrated step in the hypertrophic response (Basualto-Alarcón et al., 2013); and also through activation of G protein-linked membrane receptors, which result in calcium dependent phosphorylation of ERK1/2 (a mitogen-activated protein kinases), potentially leading to the phosphorylation of transcription factors associated with cellular growth (Kadi, 2008). However, transient activation of ERK1/2 induced by testosterone was not found to be directly related to the hypertrophic signaling cascade; though activated ERK can phosphorylate co-activators of the intracellular receptor at the nuclear level (Bratton et al., 2012), through potentiation of estrogen receptor activation function 1 (AF-1) by Src/JNK (serine 118-Independent pathway) which promotes cellular growth (Feng et al., 2001; Estrada et al., 2003). In sum, the combined effects of RE and RE-induced testosterone release induced upregulation of AR anabolism is driven via genomic and non-genomic signaling pathways which likely augment protein turnover in muscle resulting in increases in net protein accretion and hypertrophy (Wolfe et al., 2000; Roberts et al., 2018).

## Metabolic and Molecular Effects of Exercise on Oestrogen in Skeletal Muscle

Oestrogens are steroid hormones, primarily produced in the ovaries from testosterone via an aromatase enzyme, of which women have four times the amount compared with men, until the menopause (Hansen and Kjaer, 2014). While less studied in this sphere, endogenous oestrogens seem to have a metabolic role in regulating skeletal muscle; for instance, being critical for the regrowth of atrophied skeletal muscle (Sitnick et al., 2006)- an action mediated by the estrogen receptors, located within skeletal muscle tissue that function as transcription factors (Hansen and Kjaer, 2014). Indeed, as with testosterone (and perhaps as a function of its metabolic regulation through testosterone), estrogen is believed to be important in the regulation of both muscle function (Chidi-Ogbolu and Baar, 2019) and hypertrophy in response to exercise—with rapid changes in systemic concentration occurring immediately post RE, that are dependent upon RE intensity (Copeland et al., 2002), however, baseline circulating concentrations are unaltered following chronic RET for up to 6 months (Gil et al., 2012; Yoon et al., 2018). It is reported that RE acutely augments the activity of the aromatase enzyme which results in an increase in the biosynthesis of estrogen from androgens (Nelson and Bulun, 2001; Luk et al., 2015); in turn explaining the effects of RE-induced testosterone increase on an increase in estrogen levels in women (Luk et al., 2015). The effects of acute estrogen release may relate to a reduction in exercise-induced muscle damage and improved recovery (Hansen, 2018), possibly via its indirect antioxidant properties and stabilization of cell membranes (Paroo et al., 2002) and decreased post-exercise production of protein chaperones- i.e., heat shock protein (HSP) 72 (Paroo et al., 1999) and HSP70 (Enns and Tiidus, 2010). HSPs act as an index of cellular damage and activate inflammatory cell populations (e.g., neutrophils and macrophages) thereby regulating the extent of inflammatory responses after muscle injury (Senf et al., 2013). In addition, estrogen is also known to activate insulin/IGF-1 (Lee et al., 2004) and PI3K/Akt (Mangan et al., 2014) pathways, potentially enhancing the mechanisms regulating MPS (Hansen et al., 2012) and consequently muscle growth (Smith et al., 2014). The latter is suggested to occur through increased expression of Pax7 and MyoD transcription factors (Thomas et al., 2010; Sambasivan et al., 2011) which induce satellite cell expansion, differentiation, and self-renewal of muscle function and mass (Kitajima and Ono, 2016; Chidi-Ogbolu and Baar, 2019). Given that estrogen stimulates post-RE myogenesis, decreased estrogen levels in post-menopausal women may be a contributing factor to the development of sarcopenia, diminishing the rate of muscle repair and adaptive capacity in older women (Thomas et al., 2010). Indeed estrogen replacement has been shown to attenuate the age-related decline in muscle mass observed in postmenopausal women (Enns and Tiidus, 2010). However, the proposed effects of estrogen may be defined by the stage of the menstrual cycle. For example, low estrogen in the early follicular stage, may negatively affect RE-induced increases in estrogen levels (Hansen et al., 2012), while, in the luteal phase where circulating progesterone is relatively high, may also counteract the sensitizing effects of estrogen on muscle impairing any benefit of acute RE-induced during these phases (Hansen, 2018). In contrast, RET in the late part of the follicular phase, when circulating estrogen is enhanced, appears to result in increased fiber type II CSA, nuclei to fiber ratio and muscle mass, compared to RET during luteal phase (Sung et al., 2014; Wikström-Frisén et al., 2017). However, this has not been confirmed (Miller et al., 2006; Sakamaki-Sunaga et al., 2016) as no differences between follicular phase and luteal phase RET responses have also been observed, at least with regard to strength gains and hypertrophy; and as such, the role of estrogen in mediating responses to RE, remains unclear. Future trials are needed to clarify the effects of the oestrogens on muscle biology under different conditions e.g., phase of menstrual cycle, pre or post-menopause, and the response to nutrition (fasting/feeding) and exercise training (Hansen, 2018).

## Growth Hormone (GH), Exercise, and Effects on Muscle Metabolism

Human growth hormone (GH) is secreted from somatotroph cells of the anterior pituitary. It is released in 6–8 bursts.day^−1^, with negligible secretion outside of these bursts, and is under the control of the hypothalamic hormones: GH-releasing hormone (GHRH), which promotes secretion, and somatostatin which inhibits release of GH (Giustina et al., 2008). Following GH release, which induces the hepatic generation of IGF-1, circulating levels of IGF-1 and GH, feedback to the hypothalamus to inhibit further GH secretion (Daughaday, 2000). RE is the most potent physiological stimulus for GH release in both men (Nicholls and Holt, 2016; Fink et al., 2017) and women (Hymer et al., 2001), but little is known about how RE alters somatotroph content and function. GH begins to rise 10–20 min after commencing RE and peaks at the end of the RE (Gibney et al., 2007; Fink et al., 2018a), returning to baseline values around 60 min post RE (Häkkinen and Pakarinen, 1995; Fink et al., 2018a). RE increases the amplitude of each GH pulse rather than the frequency. Accordingly, whole-body RE induces GH increases from basal levels of 5 ug.L^−1^ (Fink et al., 2018b) to 24 ug.L^−1^ (Kraemer et al., 1990) while localized RE of individual muscle groups (biceps and triceps) leads to increases of only half of this; up to 12 ug.L^−1^ (Fink et al., 2018a). These increases in post RE GH levels are blunted in older adults, and a progressive decline in GH secretion and clearance is observed after the age of 40 y (Zaccaria et al., 1999). In younger adults, exercise-induced GH release is relatively non-specific occurring in response to both RE and aerobic exercise (e.g., 60% VO_2_ max) (Godfrey et al., 2003). It has also been reported that when lactate is elevated beyond anaerobic threshold, which is associated with greater demands on anaerobic glycolysis, the hypothalamus is highly stimulated (Kraemer et al., 1990, 1993; Hartman et al., 1993). This is further supported during RE protocols of moderate intensity (10-RM vs. 5-RM) with short rest periods (1 vs. 3 min) between sets (Hoffman et al., 2003), which result in higher circulating concentrations of GH in both men and women (Kraemer et al., 1993). The exact mechanism of exercise-induced GH release remain ill-defined, however are likely driven via higher intensities of RE directly stimulating the anterior pituitary, facilitated via increasing circulating of catecholamines, lactate, nitric oxide and changes in acid-base balance (Godfrey et al., 2003). Increases in post RE GH levels have also been associated with increased estrogen levels in women; i.e., area under curve (AUC) of increase in GH after RE was higher in midluteal phase than increases in early follicular phases of menstrual cycle (Nakamura et al., 2011). It seems there is a close interplay between estrogen levels and GH secretion and prevailing estrogen concentrations may modulate both GH secretion and action (Leung et al., 2004). This effect of estrogen may be due to a combination of a reduction of somatostatin's inhibitory tone, amplification of endogenous GHRH levels or its pituitary actions, and activation of additional mechanisms; e.g., estrogen stimulates GH secretion by decreasing liver secretion of IGF-1, resulting in stimulation of the pituitary to synthesize and secrete GH (Cook, 2004; Nakamura and Aizawa, 2017).

The physiological relevance of increases in GH levels after RE may be increases in protein synthesis and its ability to aid in muscle repair (Gibney et al., 2007; Liu et al., 2008) and impact on muscle mass (Hermansen et al., 2017), without any impact on muscle function (Hermansen et al., 2017). It was reported that there is a correlation between acute RE-induced GH increases and long term muscle and fiber type I and II hypertrophy (McCall et al., 1999). GH *per se* has also been associated with whole body protein synthesis (e.g., reflecting in part, connective tissues); but directly, may *not* augment MPS (Doessing et al., 2010; West et al., 2010). Conversely, acute GH infusion studies (eliciting a similar to post RE increase in GH levels) have shown this hormone may have a role in stimulating MPS (Fryburg et al., 1991; Fryburg and Barrett, 1993) while associated increases in insulin inhibit MPB (Fryburg et al., 1992). Thus, while GH is a positive regulator of extracellular matrix (ECM) synthesis (Kragstrup et al., 2011) which is important in morphogenesis (Rozario and DeSimone, 2010), there is still debate surrounding its role in the regulation of muscle mass in adults; however what may be key is the lack of effect on muscle function regardless of its impact on growth pathways and MPS.

In terms of mechanisms, following release, GH binds to its receptor leading to the recruitment and phosphorylation of Janus kinase 2 (JAK2) and its most recognized downstream target, signal transducer and activator of transcription 5 (STAT5) (Jørgensen et al., 2006). In addition, GH stimulates the IRS1/Akt (Costoya et al., 1999; Consitt et al., 2017) and mitogen-activated protein kinase (MAPK) pathways which are thought to be the main pathways contributing to GH/IGF-1-induced muscle hypertrophy via p42/p44 and p38 pathways (Consitt et al., 2017) (Figure 1). The activation of Akt results in skeletal muscle growth/maintenance since it controls the phosphorylation of a number of substrates involved in MPS including mTOR (and its downstream targets 4E-binding protein 1 (4E-BP1) and p70S6 kinase) and glycogen synthase kinase 3β (GSK3β), as well as, the inhibition of protein degradation via the forkhead transcription factor (FOXO) pathway (Consitt et al., 2017). To support these molecular effects that GH has on muscle mass, GH receptor knock-out results in a decrease in myofiber CSA and muscle mass loss in mice (Sotiropoulos et al., 2006). Nevertheless, as these mice had a reduction in circulating IGF-1 and tissue IGF-1 expression; at least in part GH dependent, it is difficult to separate the effects of the two hormones (Velloso, 2008) and further investigations are needed to clarify the main effects of GH on muscle growth in adults, in particular after RE. However, the main muscle anabolic effects of GH are believed to be indirect—via inducing the hepatic generation of IGF-1 triggering the IGF-1-Akt-mTOR pathway; in turn resulting in MPS augmentation and as a consequence muscle maintenance and growth (Sandri et al., 2013; Schiaffino et al., 2013).

## IGF-1, Exercise, and Effects on Muscle Metabolism

As previously discussed, GH acts through its receptor; however, many effects linked to RE and muscle growth are believed to act indirectly through an increase in hepatic release of IGF-1. IGF-1 can also promote muscle growth in the absence of GH; and unlike GH, IGF-1 is critical for intrauterine growth (Velloso, 2008). A liver-specific knockout mouse exhibited some postnatal growth reduction, but not as severe as with global IGF knockout (Baker et al., 1993; Tahimic et al., 2013). Bikle et al. also showed muscle atrophy was more pronounced after ablation of muscle IGF-1 production than when hepatic IGF-1 production was suppressed (Bikle et al., 2015); exhibiting circulating levels of IGF-1 (i.e., endocrine factor) do not effect overall growth responses (Ohlsson et al., 2000; Velloso, 2008). This implies that locally produced, autocrine/paracrine IGF-1 plays an important role in both pre- and postnatal growth. The local production of IGF-1 is controlled primarily by GH and other hormones (e.g., parathyroid and thyroid hormones) (Bikle et al., 2015); suggesting GH's effect on growth may be mediated in part via increased local IGF-1 production and/or action. These results indicated GH has local effects that may be independent of increased levels of the circulating IGF-I (Ohlsson et al., 2009). However, a role for circulating liver-derived IGF-I could not be excluded. Reflecting this, it has been reported that IGF-1 levels are associated with improvements in handgrip strength and physical performance as well as life-span (Birnie et al., 2012; Yusuf et al., 2020); in addition, higher circulating IGF-1 has been linked with increases in MPS, muscle free fatty acid utilization, and improvements insulin sensitivity (Kraemer et al., 2017), which may explain its often linked importance in exercise training adaptations. Systemic IGF-1 levels are rapidly increased in humans in response to RE from ~45 (resting levels) to 65 nM (immediately after RE) (Schwarz et al., 1996; West et al., 2009; Ogasawara et al., 2013) and return to baseline levels ~30 min after RE (Kraemer et al., 2017), which may play an important role for exercise-induced hypertrophy (Kido et al., 2016), neurogenesis (Trejo et al., 2001), and improved muscle strength with RET (Bjersing et al., 2017) by improving translational efficiency (Schiaffino and Mammucari, 2011) and satellite cell proliferation (Velloso, 2008) in muscle and in the central nervous system (CNS) (Mainardi et al., 2015). GH-induced IGF-1 released from the liver in response to RE is involved in two negative feedback loops. One directly affects the somatotropic cells of the anterior pituitary, itself inhibiting further release of GH, whilst the other affects GH releasing hormone and somatostatin release from the hypothalamus to reduce the secretion of GH. Repeated bouts of RE resulted in an exercise-induced GH response to each acute exercise episode, thereby increasing the 24-h secretion of GH and then IGF-1. Thus, exercise counters negative feedback and so IGF-1 secretion is maintained or increased (Godfrey et al., 2003). Further, to what we already showed [i.e., testosterone increased IGF-1 gene expression during RET (Gharahdaghi et al., 2019)], both testosterone and estrogen blunted IGF-I feedback-dependent inhibition of GH secretion (Veldhuis et al., 2004, 2005); and as it was reported in prepubertal boys, lead to increase in GH and then IGF-1 levels; which in turn exhibit the further and indirect anabolic links between androgens and muscle growth (Mauras et al., 2003). Like testosterone levels, older adults experience a lower basal level of IGF-1 (the so-called somatopause which refers to the diminishment of the GH-IGF-1 system) which attenuates post-RE levels of IGF-1 (Kraemer et al., 1999). However, RE increases IGF-1 mRNA and IGF-1 peptide production in younger adults which result in increases in muscle DNA and protein content (Adams and Haddad, 1996). Increased expression of IGF-1 in muscle leads to muscle hypertrophy in mice; which is independent of effects of circulating levels of IGF-1 (Coleman et al., 1995). Therefore, serum levels of IGF-1 (resting levels or acutely after RE) may not be a good reflection of local effects of IGF-1 (Bartke and Darcy, 2017; Van Nieuwpoort et al., 2018), especially in those tissues that have capabilities of producing the hormone themselves, such as skeletal muscle (Barclay et al., 2019). Indeed, circulating IGF-1 levels have even been shown to decrease during periods of active muscle building, likely due to a redistribution of IGF-1 from the circulation into the muscle (Arnarson et al., 2015). If such a sequestration of IGF-1 into muscle increases during RE (with a decrease in cellular GH receptors), it might occur as a result of reduced GH-induced hepatic production (Eliakim et al., 1998) and it may be speculated that the effect would be more pronounced in individuals experiencing greater activation of intracellular muscle signaling and subsequent muscle hypertrophy and performance (Velloso, 2008; Arnarson et al., 2015; Morton et al., 2016). This suggests that intrinsic secretion (i.e. autocrine) of muscle IGF-1, beside circulating IGF-1, may be a determinant for switching on anabolic pathways (Morton et al., 2018) and fusion of satellite cells (Velloso, 2008).

Given the fairly short half-life of unbound IGF-1 in serum (i.e., 5–10 min), binding to an IGF binding protein (IGFBP-3 is the most prevalent) in serum or in ECM increases IGF-1 half-life to around 25 min (Allard and Duan, 2018). In addition, IGFBPs are important in potentiating IGF-1 anabolic signaling. The potentiating action occurs when the IGF-1-IGFBP binds to the target cell's ECM components, which results in activation of IGF-1 receptor (IGFR) and then IGF-1 enters the cell and triggers phosphoinositide 3-kinase (P13-K) to generate phosphatidylinositol-bisphosphate (PIP2) (Pinedo-Villanueva et al., 2019), leading to the production of phosphatidylinositol 3,4,5-trisphosphate (PIP3) (O'Neill et al., 2015). PIP3 is then free to bind to phosphoinositide-dependent kinase-1 (PDK1) which activates the Akt-mTORC1 pathway (Schiaffino and Mammucari, 2011) promoting ribosomal biogenesis and translation to permit increases in MPS and the formation myofibrillar proteins, which allows muscle mass growth (Menon et al., 2014; Wen et al., 2016) (Figure 1). Similar to GH, IGF-1 alone stimulates the IRS1/Akt (Costoya et al., 1999; Consitt et al., 2017) and mitogen-activated protein kinase (MAPK) pathways which are thought to be main pathways contributing to GH/IGF-1-induced muscle hypertrophy (Consitt et al., 2017). Also, RE-induced IGF1-Akt activation phosphorylates AS160 (Akt substrate of 160 kDa) resulting in enhanced GLUT4 translocation and glucose uptake, reflecting the mediator role of IGF-1 in glycaemic control via insulin-IGF-1-Akt pathway activation in muscle (Kido et al., 2016). Taken together, IGF-1 signaling, including prolonged Akt and AS160 phosphorylation, may be a specific signal response to acute RE; which transduces mechanical signals leading to anabolic responses and allow IGF-1 signaling to stimulate the competing processes of muscle cellular growth.

## Conclusion

RE-induced increases in key endogenous steroid and peptide hormone responses are likely to be an integral part of the integrated response to acute exercise and exercise-induced muscle growth. The combined effects of RE and RE-induced androgen release lead to upregulation of anabolic signaling pathways which likely augment net protein accretion and hypertrophy. However, the anabolic effects of RE-induced GH release act indirectly via stimulation of hepatic-IGF-1 production; in turn resulting in the activation of anabolic signaling pathways, and muscle growth and maintenance.

Lower levels of these anabolic hormones in older adults induces anabolic resistance during RE which may partially explain their low sensitivity to a given anabolic stimulus. Hormonal patterns are obviously physiologically distinct in females and males, complicating true clarity of the isolated effects e.g., of the sex hormones (higher testosterone levels may play an important role for the adaption to RET in men; whereas in premenopausal women, estrogen may enhance the sensitivity to anabolic stimuli). Further studies are required to isolate clear hierarchical roles of the key anabolic hormones/peptides in regulating muscle growth in adults, in particular after RE, and to elucidate sex differences and their mechanisms.

## Author Contributions

NG, DW, and PA drafted the manuscript and BP, NS, KS, DW, and PA helped in literature search and edited the manuscript. All authors approved the submitted version.

## Conflict of Interest

The authors declare that the research was conducted in the absence of any commercial or financial relationships that could be construed as a potential conflict of interest.
